# Supplementary material for: Sustained-input switches for transcription factors and microRNAs are central building blocks of eukaryotic gene circuits
Source: Genome Biol. 2013 Aug 23;14(8):R85. doi: 10.1186/gb-2013-14-8-r85 (PMC4054853; doi:10.1186/gb-2013-14-8-r85)
Supplement: Additional file 5 — HTML Browsable Motif Output. Zipped folder containing all WaRSwap and FANMOD motif output, viewable in a web browser. [file gb-2013-14-8-r85-S5.ZIP › HTML_browsable_motif_output/FANMOD_ath_tair9/sigs_fanmodm-2000.pvals.heatmaps.html/motif_id_46_011101002_tftype_ath_upstream_-1000_0.html]

```
BG_MODEL = FANMOD
MOTIF_ID = 46_011101002
TF_TYPE = ath
UPSTREAM = -1000_0


PVals
FN_0.2	FN_0.4	FN_0.6	FN_0.8
dg_60.genes	0.004	0	0.86	0.525
dg_70.genes	0.004	0	0.866	0.51
dg_80.genes	0.001	0.001	0.845	0.534

ZScores
FN_0.2	FN_0.4	FN_0.6	FN_0.8
dg_60.genes	2.699	3.305	-1.089	-0.84
dg_70.genes	2.771	3.399	-1.082	-0.818
dg_80.genes	2.762	3.389	-1.041	-0.861

StDevs
FN_0.2	FN_0.4	FN_0.6	FN_0.8
dg_60.genes	30622.082	17258.636	3159.123	394.084
dg_70.genes	29874.471	16739.178	3208.268	394.175
dg_80.genes	29815.326	16850.465	3252.657	411.589
```
